# Towards visceral fat estimation at population scale: correlation of visceral adipose tissue assessment using three-dimensional cross-sectional imaging with BIA, DXA, and single-slice CT

**DOI:** 10.3389/fendo.2023.1211696

**Published:** 2023-07-11

**Authors:** Benjamin Chan, Yan Yu, Fan Huang, Varut Vardhanabhuti

**Affiliations:** ^1^ St. Catherine’s College, University of Oxford, Oxford, United Kingdom; ^2^ Snowhill Science Limited, Hong Kong, Hong Kong SAR, China; ^3^ Department of Diagnostic Radiology, The University of Hong Kong, Hong Kong, Hong Kong SAR, China

**Keywords:** visceral adipose tissue, DXA (dual-energy x-ray absorptiometry), MRI, computed tomography, correlation

## Abstract

**Background:**

In terms of assessing obesity-associated risk, quantification of visceral adipose tissue (VAT) has become increasingly important in risk assessment for cardiovascular and metabolic diseases. However, differences exist in the accuracy of various modalities, with a lack of up-to-date comparison with three-dimensional whole volume assessment.

**Aims:**

Using CT or MRI three-dimensional whole volume VAT as a reference, we evaluated the correlation of various commonly used modalities and techniques namely body impedance analysis (BIA), dual-energy x-ray absorptiometry (DXA) as well as single slice CT to establish how these methods compare.

**Methods:**

We designed the study in two parts. First, we performed an intra-individual comparison of the 4558 participants from the UK Biobank cohorts with matching data of MRI abdominal body composition, DXA with VAT estimation, and BIA. Second, we evaluated 174 CT scans from the publicly available dataset to assess the correlation of the commonly used single-slice technique compared to three-dimensional VAT volume.

**Results:**

Across the UK Biobank cohort, the DXA-derived VAT measurement correlated better (R^2^ 0.94, p<0.0001) than BIA (R^2^ 0.49, p<0.0001) with reference three-dimensional volume on MRI. However, DXA-derived VAT correlation was worse for participants with a BMI of < 20 (R^2^ = 0.62, p=0.0013). A commonly used single slice method on CT demonstrated a modest correlation (R^2^ between 0.51 – 0.64), with best values at L3- and L4 (R^2^ L3 = 0.63, p<0.0001; L4 = 0.64, p<0.0001) compared to reference three-dimensional volume. Combining multiple slices yielded a better correlation, with a strong correlation when L2-L3 levels were combined (R^2^ = 0.92, p<0.0001).

**Conclusion:**

When deployed at scale, DXA-derived VAT volume measurement shows excellent correlation with three-dimensional volume on MRI based on the UK Biobank cohort. Whereas a single slice CT technique demonstrated moderate correlation with three-dimensional volume on CT, with a stronger correlation achieved when multiple levels were combined.

## Introduction

**Figure 1:**
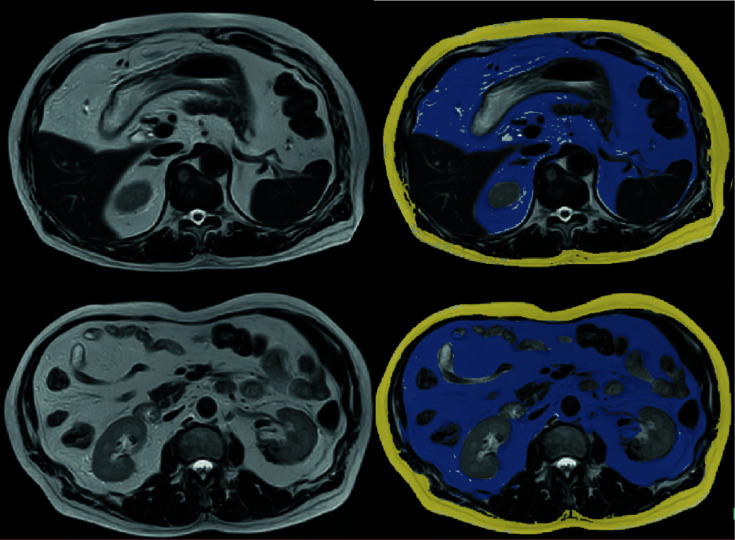
Representative examples of MRI scans with segmentation of visceral adipose tissue (blue) and subcutaneous adipose tissue (yellow).

There is a rapidly increasing demand for accurate body composition measurement particularly as we are becoming increasingly aware of the importance of visceral adipose tissue (VAT) in various diseases such as metabolic syndrome and cardiovascular diseases (1). Visceral fat is a specific compartment of fat deep within the abdominal cavity surrounding your organs (such as the liver and intestines) which must be regarded as distinct from subcutaneous fat which sits below the skin. The amount of visceral fat can vary from individual to individual and is affected by lifestyle factors such as diet and exercise as well as genetic factors. The level of body fat has been previously inferred from body weight, but we have long known that weight or body mass index is inaccurate when measuring fat. Over the past several decades, consumer devices based on body impedance analysis (BIA) technology have made measuring one’s body fat percentage more accessible to the general public. These remain great tools to track body fat, but the accuracy of these methods had previously been questioned (2–4). Looking more at clinical-based tools in healthcare settings, researchers have looked into DXA and even cross-sectional imaging to measure VAT. In clinical practice, DXA is still regarded by many as a reference standard for VAT. CT and MRI can visualise the VAT as the direct gold standard reference but is not widely available. However, this is changing more recently with the advent of deep learning technologies that have allowed for more accurate volumetric segmentation, extremely accurate visceral fat estimation on CT or MRI scans using 3-dimensional volumes rather than single slice techniques are now maturing and could potentially be adopted for more widespread use (see **Figure 1** for demonstrative example). Currently, 3 modalities are used for measuring VAT (or proxy of VAT) namely BIA, DXA and cross-sectional imaging (i.e. CT or MRI) but intra-individual comparison with reference to 3-dimensional MRI in the measurement of VF have not been assessed at a scale of 1000+ patients with all three modalities – MRI, DXA and BIA – obtained from the same subject. Furthermore, the influence of simple anthropometry measurements, e.g., body mass index (BMI), waist circumference (WC), and waist-to-hip ratio (WHR) with actual VAT has not been established using imaging and anthropometric data from the same subject on a large scale.

In the case of BIA, previous studies have compared the accuracy of BIA with DXA and MRI in body composition assessment, but these have been typically done only in a relatively small population and without the intra-subject comparison of all three modalities (3, 5). Moreover, there is limited evidence in the literature with a large sample size that investigates the influence of individual anthropometry beyond just BMI on BIA and DXA performance in VAT volume assessment.

For this study, we set out to compare at scale how these technologies perform with respect to the estimation of visceral fat. We reasoned that when available a whole volume of visceral fat on cross-sectional imaging such as CT or MRI within a subject can be regarded as a reference standard for visceral fat. We set out the study in 2 phases. First, to assess the correlation of BIA and DXA scans in the estimation of VAT with respect to the reference whole volume MRI. Within this, we also performed subanalyses comparing sex, age, country of origin, BMI, WC, and WHR on a large subject population using the UK Biobank cohorts. Second, a traditional approach of single-slice CT or MRI assessment for visceral fat is commonly used. We set out to assess the correlation between single-slice VAT assessment with reference to whole-volume VAT volume measurement.

## Materials and methods

### Participants

The study was approved by the local ethics board (UW-20814) at the University of Hong Kong. The data used were from the UK Biobank with prior consent for the patients obtained. This research has been conducted using the UK Biobank Resource under Application Number 78730. The study is set into 2 parts.

**Figure 2:**
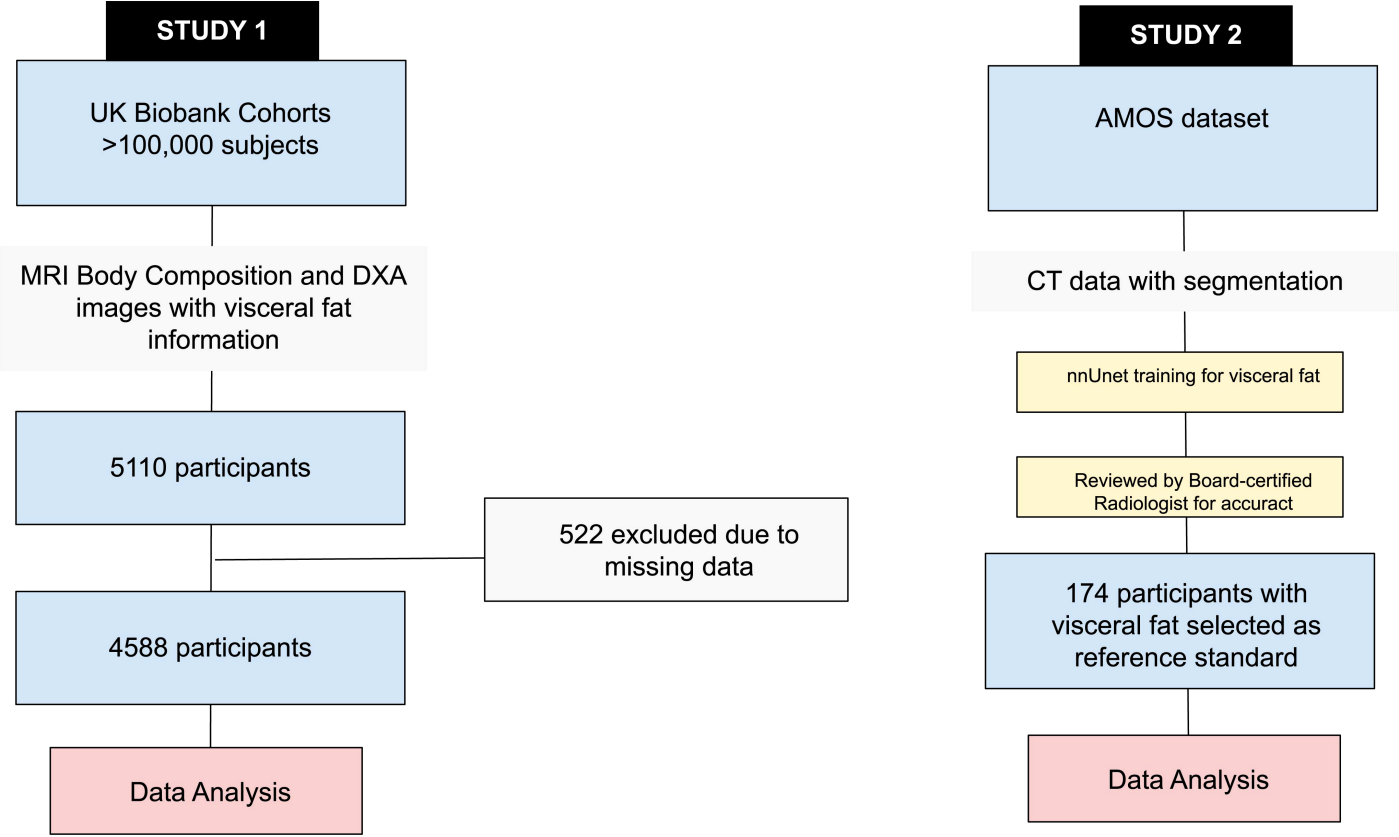
Flow chart demonstrating study design and patient cohorts for the two studies.

The first study analysed a cohort from the UK Biobank, an ongoing cohort study of individuals aged 40-69 across the UK who volunteered for the study between 2006-2010 (6). A search for participants with MRI VAT, DXA VAT and BIA measurements resulted in 5110 participants. After the exclusion of participants with missing MRI, DXA visceral fat estimation, or BIA measurement, 4588 participants remained in the analysis (please refer to the flow chart in **Figure 2**). MRI visceral fat estimation was performed based on a previously described study (7). MRI was performed at a single site (Cheadle, Stockport, UK) using Siemens 1.5T MAGNETOM Aera. DXA visceral fat estimation is a standardised method for body composition, which was previously used in a wide range of studies (8, 9). DXA was performed using Lunar iDXA (GE Healthcare, Wisconsin, USA) with proprietary body composition analysis in a single UK Biobank imaging centre (Cheadle, Stockport, UK). Numerical values were exported to the UK Biobank server without further processing. For BIA measurements, the Tanita BC418MA body composition analyser (Tanita Corporation, Arlington Heights, IL, USA) was used throughout the UK Biobank enrolment centres. Trunk fat mass and whole-body fat mass were used as proxies for body fat in the body as dedicated visceral fat estimation was not available. The estimation was done based on the prediction equation described previously based on a four compartments model (10). It must be noted that the BIA measurement of trunk fat mass and whole-body fat mass is not the same nor was intended for measuring VAT. The analysis is included here for comparison purposes as it is a technique that is widely used and adopted. Other BIA-related parameters were also analysed for comparison.

The second study used a cohort from AMOS, a database of abdominal multi-organ segmentation conducted with CT and MRI scans (11). 174 participants with a re-annotated complete set of VAT volumes at each lumbar level and whole-body VAT volume were analysed. No participant characteristics are available from AMOS. For this dataset, since whole volume visceral fat segmentation was not available, we separately trained in-house a deep learning-based model based on nnU-Net architecture (12), with generated segmentation then reviewed and amended, by a board-certified radiologist (VV with 15 years of experience), and used as the reference standard.

### Statistical analysis

All statistical analysis was performed using SPSS software (IBM Corp. Released 2022. IBM SPSS Statistics for Mac, Version 29.0.00. Armonk, NY: IBM Corp).

All significance tests were two-tailed, and p < 0.05 was considered statistically significant. For the strength of the association, for absolute values of R and R^2^, 0-0.19 is regarded as very weak, 0.2-0.39 as weak, 0.40-0.59 as moderate, 0.6-0.79 as strong and 0.8-1 as very strong correlation (13).

For the first objective, a further sub-analysis was performed based on gender, age group, country of origin, BMI, WC, and WHR to see if DXA VAT volume and BIA Whole Body and Trunk Fat Mass performance varied across these subgroups. The World Health Organisation standards for BMI and WHR stratification were applied: with BMI, BMI <18.5 means underweight, 18.5 =< BMI <25 means healthy, 25=< BMI <30 means overweight, BMI >30 means obese; with WHR, males were healthy with WHR=<0.9 and had abdominal obesity when WHR>0.9, while females were healthy with WHR=<0.85, and had abdominal obesity when WHR>0.85.

For the second objective, Pearson’s correlation was calculated between reference whole-body VAT volume estimation and the cross-sectional VAT volume obtained by CT.

## Results

A total of 4588 participants were finally included in the study (male n = 2188, 47.69%), with an average age (62.54 years for males, and 61.17 years for females). The subject characteristics including demographics, anthropometry and VAT measurements are summarised in **Table 1**.

**Table 1:** Subject characteristics of cohort from UK Biobank.

	Total		Male		Female	
**Participants**	4588		2188	47.69%	2400	52.31%
** **	Mean	SD	Mean	SD	Mean	SD
**Age**	55.57	7.56	56.3	7.53	54.9	7.52
Country of Origin	Number	% of Total	Number	% of Total	Number	% of Total
British	4178	91.06%	1999	91.36%	2179	90.79%
Other ethnic group	410	8.94%	189	8.64%	221	9.21%
Anthropometry	Mean	SD	Mean	SD	Mean	SD
Height	169.82	9.47	176.97	6.59	163.31	6.54
Weight	76.41	14.79	84.16	13.03	69.34	12.58
WC	87.45	11.94	93.46	9.62	81.97	11.2
HC	101.3	8.39	101.65	6.95	100.99	9.5
WHR	0.86	0.8	0.92	0.05	0.81	0.06
BMI	26.65	4.24	27.13	3.73	26.22	4.61
VAT measurements	Mean	SD	Mean	SD	Mean	SD
MRI	3.72	2.23	4.94	2.28	2.61	1.47
DXA	1276.2	948.73	1781.74	993.78	815.32	609.46
BIA Whole body	24.05	8.42	22.11	7.33	25.82	8.94
BIA Trunk	13.51	4.71	13.95	4.53	13.1	4.82

**Figure 3:**
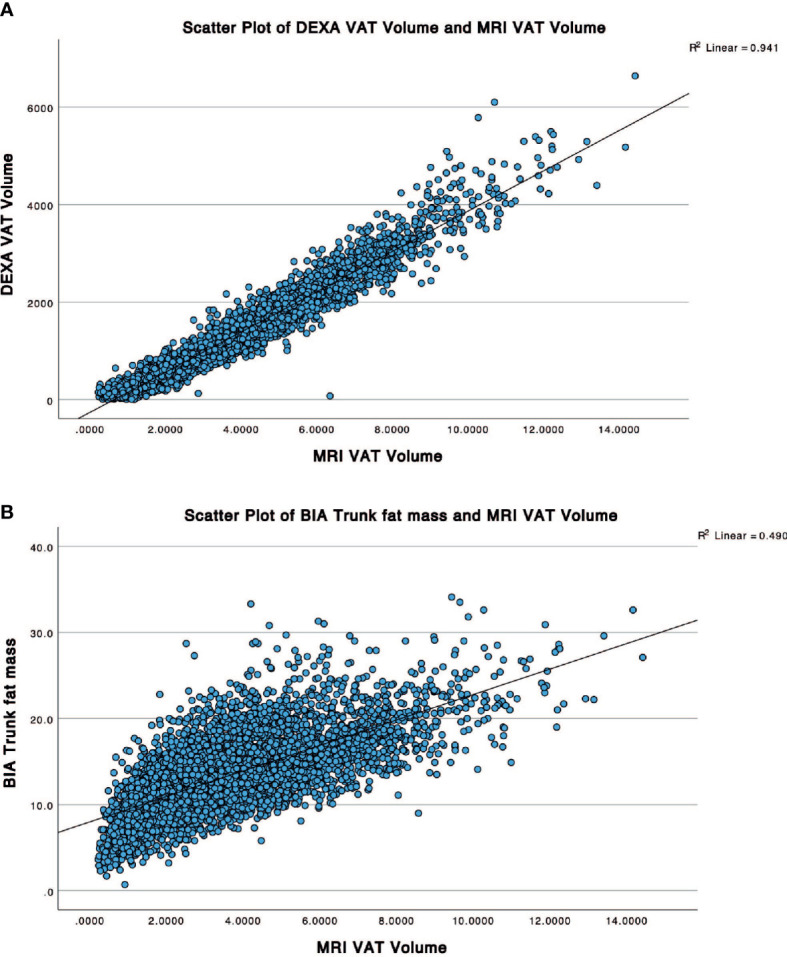
Scatter plots showing correlation between the reference MRI VAT volume with **(A)** DXA VAT Volume and **(B)** BIA trunk fat mass.

For the first part of the study, the R^2^ of DXA and BIA whole-body VAT volume measurement for all groups is summarised in **Table 2**. Across all groups, DXA-derived VAT volume measurement consistently correlated better with that of MRI (R^2^ 0.94, p<0.0001) than BIA whole-body fat mass (R^2^ 0.27, p<0.0001), and BIA trunk fat mass (R^2^ 0.49, p<0.0001). For a graphical representation of the linear correlation plots, please refer to **Figure 3**. It was also worthy of note that conventional metrics such as waist circumference (WC) demonstrated a very strong correlation with MRI VAT (R^2^ 0.826, p<0.0001), whilst BMI was less strong (R^2^ 0.672, p<0.0001).

**Table 2:** Pearson correlation using MRI VAT across different modalities of fat and body composition measurements .

DXA Parameters	Pearson Correlation	BIA Parameters	Pearson Correlation	Conventional Parameters	Pearson Correlation
DXA Volume VAT volume	0.94^**^	BIA Trunk fat percentage	0.12^**^	Waist circumference	0.68^**^
DXA Mass VAT mass	0.94^**^	BIA Trunk fat mass	0.49^**^	Weight	0.64^**^
		BIA Trunk fat-free mass	0.39^**^	Body mass index (BMI)	0.45^**^
		BIA Trunk predicted mass	0.39^**^	Hip circumference	0.27^**^
		BIA Body fat percentage	0.01^**^		
		BIA Whole body fat mass	0.27^**^		
		BIA Whole body fat-free mass	0.45^**^		
		BIA Whole body water mass	0.45^**^		

**. Correlation is significant at the <0.01 level (2-tailed).

Upon subanalysis (see **Table 3**), there is no significant difference between sexes when using DXA (R^2^ male 0.93, p<0.0001; female 0.91, p<0.0001), but BIA performed better in males. DXA performance was consistent across age groups, while BIA correlation improved with increasing age. There is no significant difference in correlation in DXA and BIA correlation between British (R^2^ = 0.94, p<0.0001) and non-British populations (R^2^ = 0.94, p<0.0001). For differences in body anthropometry, DXA was highly consistent except for at low BMI. Notably, DXA correlation was worse for participants with low BMI (< 20) (R^2^ = 0.62, p=0.0013); an arbitrary threshold of BMI < 20 was applied to increase group size, due to the small sample size (n = 31) of those that satisfy the WHO criteria of being underweight (BMI<18.5). DXA correlated better with higher BMI (R^2^ for normal (0.89), overweight (0.92), and obese (0.91), respectively). BIA performance was inconsistent with BMI and demonstrated a weak to moderate correlation with WC (range 0.19-0.56).

**Table 3:** Correlation analysis (R^2^) using three dimension MRI visceral adipose tissue as reference comparing DXA and BIA with subanalysis based on different participants demographics and characteristics.

	n	DXA R^2^	p-value	BIA (Whole-body fat mass) R^2^	p-value	BIA (Trunk fat mass) R^2^	p-value
**Total**	4588	0.94	<0.0001	0.27	<0.0001	0.49	<0.0001
Sex							
Male	2188	0.93	<0.0001	0.67	<0.0001	0.67	<0.0001
Female	2400	0.91	<0.0001	0.62	<0.0001	0.58	<0.0001
Age							
40-49	1128	0.95	<0.0001	0.26	<0.0001	0.44	<0.0001
50-59	1822	0.94	<0.0001	0.27	<0.0001	0.48	<0.0001
60-69	1630	0.94	<0.0001	0.31	<0.0001	0.56	<0.0001
Country of Origin							
British	4178	0.94	<0.0001	0.49	<0.0001	0.27	<0.0001
Non-British	410	0.94	<0.0001	0.45	<0.0001	0.24	<0.0001
BMI							
Underweight (<18.5)	31	0.30	0.0013	0.16	0.0254	0.15	0.0287
Low (<20)**	128	0.62	<0.0001	0.05	0.00871	0.12	<0.0001
Normal (18.5=<BMI<25)	1713	0.89	<0.0001	0.05	<0.0001	0.25	<0.0001
Overweight (25=<BMI<30)	1973	0.92	<0.0001	0.00	0.287	0.15	<0.0001
Obese (BMI>=30)	871	0.91	<0.0001	0.00	0.216	0.18	<0.0001
WC							
Healthy male (WC<102)	1795	0.92	<0.0001	0.58	<0.0001	0.56	<0.0001
Obese male (WC>=102)	393	0.82	<0.0001	0.32	<0.0001	0.34	<0.0001
Healthy female (WC<90)	1840	0.87	<0.0001	0.44	<0.0001	0.40	<0.0001
Obese female (WC>=90)	560	0.78	<0.0001	0.23	<0.0001	0.19	<0.0001
WHR							
Healthy male (WHR=<0.9)	816	0.90	<0.0001	0.60	<0.0001	0.58	<0.0001
Abdominal obesity male (WHR>0.9)	1372	0.91	<0.0001	0.58	<0.0001	0.58	<0.0001
Healthy female (WHR=<0.85)	1757	0.88	<0.0001	0.60	<0.0001	0.57	<0.0001
Abdominal obesity female (WHR>0.85)	643	0.86	<0.0001	0.52	<0.0001	0.49	<0.0001

* 8 subjects were between 70-77 years of age. However, due to the small number of those in the 70+ age group, these subjects were excluded from the age-group analysis.

** An arbitrary threshold for low BMI (<20) was applied to increase group size, due to the small sample size (n = 31) of those that satisfy the WHO criteria of being underweight (BMI<18.5).

Another common method to assess visceral fat is to use a single-slice CT examination. To investigate the second objective, we assessed the correlation between whole-body VAT volume estimate with CT-derived VAT volume measurement along lumbar cross-sections. A conventional method for assessing visceral fat on CT has been to perform this on a single slice. Here we set out to investigate the correlation at different levels and in combinations with the reference standard being the whole volume segmentation. The results are summarised in **Table 4**. When a single slice method was used, this demonstrated a modest correlation (R^2^ between 0.51 – 0.64), with best values at L3- and L4 (R^2^ L3 = 0.63, p<0.0001; L4 = 0.64, p<0.0001). When a few slices were combined at a single vertebra level, these demonstrate better performance (R^2^ between 0.63 – 0.81), with the best value at the L3 vertebra. As expected, when multi-level combinations were done, this resulted in improved correlation, for example, combining all CT scans of the entire lumbar spine L1-L5 (R^2^ = 0.97, p<0.0001), but very strong correlation when only 2 levels were combined, for example at L2-L3 (R^2^ = 0.92, p<0.0001).

**Table 4:** Correlation analysis of single slice and multi-slice combination compared with three dimension whole volume visceral adipose tissue in the AMOS datasets. .

Single Slice	L1 mid-slice	L2 mid-slice	L3 mid-slice	L4 mid-slice	L5 mid-slice					
**R^2^**	0.51	0.62	0.63	0.64	0.47					
**p-value**	<0.0001	<0.0001	<0.0001	<0.0001	<0.0001					
Single Vertebra Volume	**L1**	**L2**	**L3**	**L4**	**L5**					
**R^2^**	0.63	0.79	0.81	0.78	0.75					
**p-value**	<0.0001	<0.0001	<0.0001	<0.0001	<0.0001					
Combination	**L1-L2**	**L1-L3**	**L1-L4**	**L1-L5**	**L2-L3**	**L2-L4**	**L2-L5**	**L3-L4**	**L3-L5**	**L4-L5**
**R^2^**	0.83	0.92	0.95	0.97	0.92	0.94	0.96	0.9	0.94	0.91
**p-value**	<0.0001	<0.0001	<0.0001	<0.0001	<0.0001	<0.0001	<0.0001	<0.0001	<0.0001	<0.0001

## Discussion

Two assessments were investigated in this study. First, the correlation of BIA and DXA VAT volume measurement for the estimation of visceral fat with reference whole volume MRI using the UK Biobank cohort demonstrated consistently high correlation using DXA-derived visceral fat, but only weak to moderate correlation using BIA whole-body fat mass and trunk fat mass. We also made a comparison with conventional parameters such as WC and BMI for comparison. Second, CT-derived VAT estimation demonstrated moderate correlation using a commonly utilised single-slice technique. To our knowledge, this is the first study that assesses the accuracy of all three body composition modalities – BIA, DXA and MRI – with intra-individual comparison in a large population and explored the correlation with commonly used techniques using a single slice CT at multiple levels and combinations.

In relation to the first objective, our study demonstrated that DXA is extremely accurate and correlates very closely with whole-body VAT volume obtained from MRI. This is despite the known variability between different vendors in estimating VAT as well as variability related to hydration status, age and gender. Despite the potential shortcomings of DXA, and also to the investigators’ surprise, it was demonstrated that there was little variability between sex, age group, country of origin, and WHR but notably, DXA performed poorly in subjects with low BMI, while the correlation was slightly diminished in those with high WC. Therefore, it may be worth considering the BMI and WC of patients when making clinical interpretations of VAT volume using DXA. On the other hand, whole-body and trunk fat estimation obtained from BIA correlated relatively poorly with MRI visceral fat and showed larger variability. It must be noted that the BIA estimation used in this study was not designed to estimate visceral fat. Moreover, there is likely variability between different vendors in terms of their accuracy (owing to the use of different proprietary algorithms) to be able to make a meaningful comparison at a population scale. Although, BIA using the same machine can be useful to monitor and track longitudinally, in this study, we demonstrated that it is a poor estimator for visceral fat which is a more important metric to monitor and track clinically. Performance for BIA was especially poor when subjects were segregated by BMI and age, showing poor consistency. Considering the poor correlation of BIA in VAT estimation, it should be used in the knowledge that this is an estimator of body fat, which has a poor correlation with visceral fat, despite the advantage of cost and accessibility as an alternative to DXA and MRI in body composition analysis. We acknowledge that some BIA devices can employ specific algorithms to estimate whole-body VAT (14), but these were not available in the current study and therefore were not directly compared.

In relation to our second objective, CT-derived VAT estimation demonstrated a moderate correlation with reference whole volume assessment. With the advent of deep learning, where automatic segmentation of organs including visceral fat is now maturing, a more accurate method for quantifying visceral fat may be available for more widespread use and should be taken as the gold standard method for quantifying visceral fat when available.

There are some limitations worth noting. First, it must be noted that the various imaging techniques are measuring VAT differently. For example, anatomically DXA and MRI are measuring VAT in slightly different anatomical regions and also in different units. The extrapolation from this study is for agreement as assessed by correlation and we acknowledge that despite a strong correlation, there may be some bias if they are measuring 2 different entities. Second, we did not compare the impact on other population groups. The UK biobank cohort contains a predominantly British Caucasian population. However, as the study focuses on intra-individual comparison, the impact of this is thought to be minimal. Third, we acknowledge that BIA and DXA are affected by various external and internal factors that might influence their accuracy. For example, various BIA algorithms were validated mainly on individuals with stable water and electrolytes balance and are dependent on age, sex and race (15). It is affected by skin temperature (16). It has been shown that the use of general BIA equations across different ethnic groups results in bias (17). Meanwhile, water intake and hydration status impact the muscle compartments and may lead to inaccuracies in fat estimation in DXA.

In conclusion, we have demonstrated a strong correlation of DXA-derived VAT when assessing at the population scale as applied to the UK Biobank cohort whilst BIA total and trunk body fat mass demonstrate a moderate correlation for three-dimensional VAT. Similarly, a single-slice technique in CT demonstrated moderate correlation. Future whole-body CT or MRI automated segmentation of visceral fat may become more readily available but needs further validation if this could amount to more accurate biomarkers for risk stratification.

## Data availability statement

The data analysed in this study is subject to the following licenses/restrictions: The data from this study is accessible via application to the UK Biobank Study. Requests to access these datasets should be directed to UK Biobank https://bbams.ndph.ox.ac.uk/ams/resApplications.

## Ethics statement

The studies involving human participants were reviewed and approved by University of Hong Kong Institutional Review Ethics Board (US-20814). The data used were from the UK 212 Biobank with prior consent for the patients obtained.

## Author contributions

VV, YY, and BC contributed to the conception and design of the study. VV and BC retrieved data from UK Biobank. VV and FH retrieved data from AMOS. FH performed AMOS data analysis. BC and VV performed the statistical analysis for all parts. BC wrote the first draft of the manuscript. VV, YY, and FH edited and contributed to sections of the manuscript. All authors contributed to manuscript revision, read, and approved the final submitted version.
