# Supplementary material for: Towards visceral fat estimation at population scale: correlation of visceral adipose tissue assessment using three-dimensional cross-sectional imaging with BIA, DXA, and single-slice CT
Source: Front Endocrinol (Lausanne). 2023 Jul 11;14:1211696. doi: 10.3389/fendo.2023.1211696 (PMC10368369; doi:10.3389/fendo.2023.1211696)
Supplement: Supplementary file 1 [file Table_1.docx]

**Supplementary Table 1**

* 8 subjects were between 70-77 years of age. However, due to the small number of those in the 70+ age group, these subjects were excluded from the age-group analysis.

** An arbitrary threshold for low BMI (<20) was applied to increase group size, due to the small sample size (n = 31) of those that satisfy the WHO criteria of being underweight (BMI<18.5).

|  | n | DXA R-value | p-value | BIA (Whole-body fat mass) R-value | p-value | BIA  (Trunk fat mass) R-value | p-value |
| --- | --- | --- | --- | --- | --- | --- | --- |
| **Total** | 4588 | 0.970  (0.968-0.972) | <0.0001 | 0.521  (0.500-0.542) | <0.0001 | 0.700  (0.685-0.714) | <0.0001 |
|  |  |  |  |  |  |  |  |
| **Sex** |  |  |  |  |  |  |  |
| Male | 2188 | 0.963  (0.960-0.966) | <0.0001 | 0.818  (0.803-831) | <0.0001 | 0.816  (0.802-0.830) | <0.0001 |
| Female | 2400 | 0.952  (0.948-0.956) | <0.0001 | 0.788  (0.772-0.802) | <0.0001 | 0.761  (0.744-0.778) | <0.0001 |
|  |  |  |  |  |  |  |  |
| **Age*** |  |  |  |  |  |  |  |
| 40-49 | 1128 | 0.974  (0.970-0.977) | <0.0001 | 0.506  (0.462-0.549) | <0.0001 | 0.661  (0.627-0.693) | <0.0001 |
| 50-59 | 1822 | 0.969  (0.966-0.972) | <0.0001 | 0.521  (0.486-0.553) | <0.0001 | 0.693  (0.688-0.716) | <0.0001 |
| 60-69 | 1630 | 0.968  (0.965-0.971) | <0.0001 | 0.553  (0.518-0.586) | <0.0001 | 0.745  (0.723-0.766) | <0.0001 |
|  |  |  |  |  |  |  |  |
| **Country of Origin** |  |  |  |  |  |  |  |
| British | 4178 | 0.970  (0.968-0.972) | <0.0001 | 0.524  (0.502-0.546) | <0.0001 | 0.703  (0.687-0.718) | <0.0001 |
| Non-British | 410 | 0.970  (0.964-0.975) | <0.0001 | 0.489  (0.412-0.559) | <0.0001 | 0.671  (0.614-0.721) | <0.0001 |
|  |  |  |  |  |  |  |  |
| **BMI** |  |  |  |  |  |  |  |
| Underweight (<18.5) | 31 | 0.550  (0.243-0.757) | 0.0013 | 0.401  (0.055-0.662) | 0.0254 | 0.393  (0.045-0.656) | 0.0287 |
| Low (<20)** | 128 | 0.787  (0.710-0.845) | <0.0001 | 0.231  (0.060-0.389) | 0.00871 | 0.347  (0.185-0.491) | <0.0001 |
| Normal (18.5=<BMI<25) | 1713 | 0.945  (0.940-0.950) | <0.0001 | 0.218  (0.173-0.263) | <0.0001 | 0.500  (0.464-0.535) | <0.0001 |
| Overweight (25=<BMI<30) | 1973 | 0.957  (0.945-0.957) | <0.0001 | 0.024  (-0.020-0.068) | 0.287 | 0.393  (0.355-0.430) | <0.0001 |
| Obese (BMI>=30) | 871 | 0.952  (0.945-0.957) | <0.0001 | 0.042  (-0.024-0.109) | 0.216 | 0.425  (0.369-0.478) | <0.0001 |
|  |  |  |  |  |  |  |  |
| **WC** |  |  |  |  |  |  |  |
| Healthy male (WC<102) | 1795 | 0.958  (0.954-0.962) | <0.0001 | 0.762  (0.742-0.780) | <0.0001 | 0.750  (0.729-0.769) | <0.0001 |
| Obese male (WC>=102) | 393 | 0.905  (0.886-0.922) | <0.0001 | 0.562  (0.490-0.626) | <0.0001 | 0.581  (0.511-0.643) | <0.0001 |
| Healthy female (WC<90) | 1840 | 0.934  (0.928-0.940) | <0.0001 | 0.662  (0.636-0.687) | <0.0001 | 0.631  (0.602-0.657) | <0.0001 |
| Obese female (WC>=90) | 560 | 0.885  (0.866-0.902) | <0.0001 | 0.481  (0.415-0.542) | <0.0001 | 0.432  (0.363-0.498) | <0.0001 |
|  |  |  |  |  |  |  |  |
| **WHR** |  |  |  |  |  |  |  |
| Healthy male (WHR=<0.9) | 816 | 0.951  (0.944-0.957) | <0.0001 | 0.774  (0.745-0.800) | <0.0001 | 0.763  (0.733-0.790) | <0.0001 |
| Abdominal obesity male (WHR>0.9) | 1372 | 0.952  (0.947-0.957) | <0.0001 | 0.760  (0.737-0.782) | <0.0001 | 0.761  (0.738-0.783) | <0.0001 |
| Healthy female (WHR=<0.85) | 1757 | 0.940  (0.934-0.945) | <0.0001 | 0.777  (0.758-0.795) | <0.0001 | 0.754  (0.733-0.773) | <0.0001 |
| Abdominal obesity female (WHR>0.85) | 643 | 0.930  (0.919-0.940) | <0.0001 | 0.722  (0.683-0.757) | <0.0001 | 0.699  (0.657-0.736) | <0.0001 |
